# Polymicrobial Necrotizing Fasciitis in a Dog: The Involvement of *Macrococcus caseolyticus*, *Proteus mirabilis*, and *Escherichia coli*

**DOI:** 10.1155/2021/5544558

**Published:** 2021-03-30

**Authors:** Obed Danso Acheampong, Ben Enyetornye, Dominic Osei

**Affiliations:** School of Veterinary Medicine, College of Basic and Applied Sciences, University of Ghana, P.O. Box LG139, Legon-Accra, Ghana

## Abstract

A male mixed breed dog was presented with two large wounds, extending the epidermis, dermis, and fascia: one at the dorsum of the thoracolumbar region and the other at the lumbosacral area. Lesions had extended inconspicuously to the dorsum of thorax affecting a large area, which showed regions with necrotic and crepitating foci after shaving. Based on histopathological and bacterial culture examinations, polymicrobial necrotizing fasciitis (NF) was diagnosed. Using the Bruker MALDI Biotyper identification technique, *Macrococcus caseolyticus*, *Proteus mirabilis*, *and Escherichia coli* were identified. Hitherto, there is no report on these bacteria linking them simultaneously to NF in a dog. In addition, the authors highlight other microbes associated with NF in humans and animals.

## 1. Introduction

Necrotizing fasciitis (NF) is a rare, severe, and extensive infection with sudden onset and characterized by, in most cases, sudden progressive necrosis involving all skin layers, fascia, and underlying soft tissues [1, 2]. NF is often associated with intense pain [3], ulcerative necrosis of affected parts of the body, systemic signs of shock [1, 4], and death [2]. Initial clinical signs include localized swelling, erythema, and warmth [1, 3]. Within hours, the infection may progress from the nidus to affect large areas of the skin and underlying tissues [1]. This case report demonstrates three different bacteria identified in a single NF case in a dog, which until now is yet to be reported. This report discusses further other microbes linked to NF in humans and animals and accentuates the relevance of microbial identification in all cases of NF. Additionally, the authors describe the histopathological and dermatological changes that were associated with this case.

## 2. Case Details

**Figure 1:**
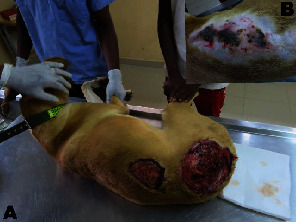
The patient on an examination bench. (a) Two large deep ulcers on the dorsum spanning the thoraco-lumbosacral region with cellulitis. (b) The dorsum of the thorax, after shaving, showing coalescing multifocal purple to black necrotic skin.

A 5-year-old intact male mixed breed dog (body weight = 28.0 kg) was presented with two wide dorsal ulcers: the dimension of the thoracolumbar lesion was 10.0 cm × 9.0 cm and the circular lumbosacral lesion a diameter of 15.8 cm (Figure 1(a)). Overlying skin of the affected areas sloughed off, according to history, exposing a crust-coated reddened subcutaneous and deep fascia tissue (Figure 1(a)). History also indicated no antimicrobial or prolonged immunosuppressive drug therapy. Affected areas were highly sensitive to touch, inciting intense painful reactions from the patient. The patient was depressed, lethargic, hyperpneic, and feverish (40.2°C). After clinical examination and shaving of the dorsal thorax, this area showed crepitating cyanotic patches of necrotizing skin (Figure 1(b)).

Using 13 × 150 mm sterile swab sticks (Hangzhou Co. Ltd., China), deep wound swabs were obtained for bacterial culture and sensitivity testing; afterwards, skin biopsy was taken for histopathology. Complete blood count depicted leukocytosis characterized by (i) marked monocytosis, indicating tissue necrosis; (ii) intense neutrophilia, indicative of stress or infection; and (iii) mild basophilia (Table 1). The overall leukocyte pattern suggested an acute inflammatory response with tissue necrosis. Serum biochemistry analysis was unremarkable, except for hyperbilirubinemia, mild hyponatremia, mild hypochloremia, and increased ALP and AST levels (Table 2).

WBC: white blood cell; PLT: platelet; RBC: red blood cell; HGB: hemoglobin; HCT: hematocrit; MCV: mean corpuscular volume; MCH: mean corpuscular hemoglobin; MCHC: mean corpuscular hemoglobin concentration; RDW: red cell distribution width.

AST: aspartate aminotransaminase; ALT: alanine aminotransaminase; ALP: alkaline phosphatase; GGT: gamma glutamyl transferase.

With bacterial culture and histopathology results pending, treatment of the patient commenced. Wounds were debrided and clean-disinfected under general anesthesia (xylazine, 2.2 mg/kg; plus ketamine 3 mg/kg; Sigma-Aldrich, India); and during the procedure, the patient received a polyionic isotonic fluid (Ringer's lactate IV; Nirlife-Aculife, Sachana, India) at a rate of 1.8 mL/kg/h IV. Amoxicillin, (20 mg/kg) (Interchemie, Venray, Holland) and enrofloxacin (5 mg/kg) (Tradeon Band, Dombivli, India) were administered, all IM, and metamizole 30 mg/kg IV (Sigma-Aldrich, India) for analgesia, postdebridement. Unceremoniously, the dog was euthanized the following day at the request of the owner.

Nonetheless, using the Bruker MALDI Biotyper, *Macrococcus caseolyticus* (score value: 1.84), *Proteus mirabilis* (score value: 2.14), and *Escherichia coli* (score value: 2.22) were later identified (Table 3).

Tet: tetracycline; Cot: cotrimoxazole; Crx: cefuroxime; Gen: gentamycin; Ery: erythromycin; Flx: flucloxacillin; Pen: penicillin; Amp: ampicillin; R: resistant; +: intermediate susceptibility, results are equivocal; ++: moderately susceptible, may be inhibited provided a higher dosage is used; +++: susceptible, infection may respond to treatment at normal dosage.

**Figure 2:**
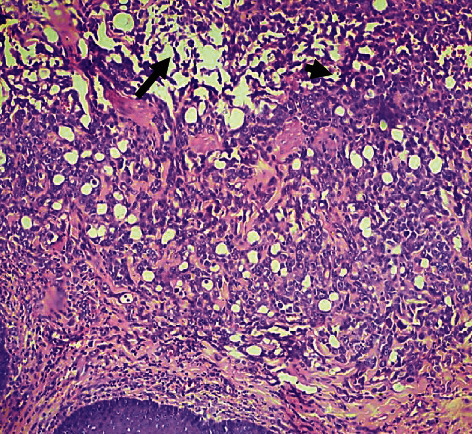
Photomicrograph of the skin (haematoxylin and eosin stain, ×100) showing damaged superficial fascia and subcutaneous tissue (arrow), infiltrated by necrotic and effete polymorphs and histiocytes (arrowhead) and bacteria.

**Figure 3:**
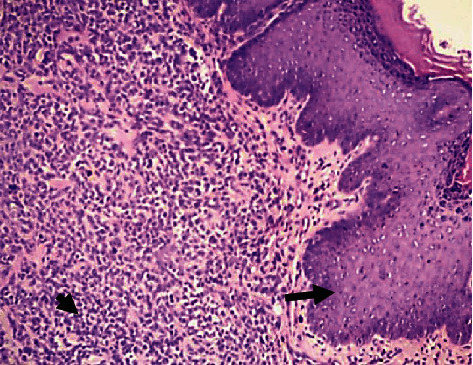
Photomicrograph (haematoxylin and eosin stain, ×100) demonstrating epidermal hyperplasia (arrow) and mass infiltration of the subcutis and dermis with bacteria and inflammatory cells (arrowhead).

Histopathology demonstrated superficial fascia necrosis, mild dermal fibrosis, orthokeratotic and parakeratotic hyperkeratosis, moderate histiocytic and polymorphonuclear cell infiltration of the dermis and fascia, and presence of bacteria within the damaged fascia and dermis (Figures 2 and 3).

## 3. Discussion

Necrotizing fasciitis usually has a polymicrobial etiology; notwithstanding, monomicrobial NF also occurs and is less common, mostly amongst healthy patients with a history of trauma. Polymicrobial NF occurs in individuals with preexisting conditions such as diabetes, immunosuppressive drugs, malnutrition, senility, malignancy, renal failure, obesity, and other chronic diseases [5]. Dissemination of infection is facilitated by activities of bacterial toxins and tissue-damaging enzymes, which cause severe subcutaneous damage, often with little or no overt changes in the overlying skin. These processes account for the high thresholds of pain associated with NF [3]. The extent of pain expression in affected dogs and humans is exaggerated vis-à-vis the physical appraisal of the lesions [1, 3]. Primarily, early diagnoses of NF improve prognoses and ensure successful therapeutic outcomes [1].

The anamnesis of this dog indicated no skin trauma; however, it is not seldom for NF to occur with no perceptible breaks in skin integrity [6]. Bites, ranging from minor to severe types, may easily introduce bacteria into the skin, which may cause NF. Early symptoms mimic those of cellulites, but advanced skin changes such as skin ulceration, formation of bullae, gas accumulation in tissues, and fluid draining may occur rapidly as the infection progresses. Hemorrhagic bullae and crepitus are sinister signs, with the likelihood of underlying fascia and muscle being compromised [5]. In this case, crepitus was felt over the affected areas of the thorax (Figure 1(b)). Histopathology and bacterial culture are required for definitive diagnosis [7]. The sudden onset of NF in this dog, along with the extensive tissue damage, explains the observed leucocyte pattern, characterized by marked neutrophilia and monocytosis (Table 1). Paucity of cutaneous manifestations, especially in dogs with thick hair coats, in the early course of NF makes diagnosis a daunting task. Early diagnosis of NF and treatment with extensive excision and debridement, in addition to a suitable antimicrobial therapy, are the key to reducing mortality [5]. Hence, a presumptive suspicion of NF warrants the commencement of treatment, even when laboratory test results are pending [7]. Immediate and aggressive surgical debridement is essential in abating morbidity and mortality [1].

Bacterial culture and isolation test results from affected dogs often demonstrate polymicrobial NF. Anaerobes are commonly isolated [3, 6, 8]. *Staphylococcus pseudintermedius* and *Streptococcus canis* are the well-known facultative anaerobes associated with NF in dogs [6, 9]. *S. pseudintermedius*, the main cause of NF in dogs, has genetic similarities to isolates in human cases, which is telling of its zoonotic potential [6]. Interestingly, our investigations using the Bruker MALDI Biotyper revealed *Macrococcus caseolyticus*, *Proteus mirabilis*, and *Escherichia coli* as the cause of NF in this patient (Table 3). *Proteus mirabilis* is an aerobic gram-negative bacterium, ubiquitous, and often isolated from urine, throat, and fecal samples [10]. *Escherichia coli*, a gram-negative facultative anaerobic bacterium, is mainly a gut microbe [11]. *M. caseolyticus* is an aerobic gram-positive bacterium and has been isolated from different mammals, even from milk and meat products [12, 13]. *M. caseolyticus* belongs to the normal skin flora of most domestic animals, methicillin-resistant, and causes mastitis in cows [13, 14]. Until now, there is no report of *E. coli* involvement in NF cases in dogs. *P. mirabilis* is famous for urinary tract infections, otitis, and diarrhea and poses great public health risks [10]; interestingly, this bacterium has not been reported in literature in association with NF cases in dogs. Also worth noting is the possibility that *P. mirabilis* was a coincidental finding or a contaminant. However, cases of NF in humans have been linked to *P. mirabilis* [15, 16], unlike in dogs. *M. caseolyticus*, a catalase- and oxidase-positive bacterium, is phylogenetically closely related to the genus *Staphylococcus*. Despite the low pathogenic potential of *M. caseolyticus*, it has been associated with abscesses in lambs and, lately, as the causative agent of broiler chicken infections [13]. At the time of writing this report, *M. caseolyticus* has been linked only to an ear skin infection in a dog [17].

Other microbes have been linked to NF in both humans and animals. Toxigenic strains of *Corynebacterium ulcerans*, isolates from dogs, have been associated with severe NF in human patients [18]. *Mucor indicus*, a zygomycete fungus, caused NF in a pediatric bone marrow transplant recipient [19]. *Streptococcus agalactiae* also caused an NF outbreak in a group of juvenile saltwater crocodiles, *Crocodylus porosus* [4]. A human patient suffered NF, caused by *E. coli*, after renal transplantation [20]. Also, out of 45 reviewed NF cases at an infectious disease unit in Israel, 22% of them were caused by *E. coli* [11], which has not been so far linked to cases in dogs. According to Sasaki et al., 2017's report, methicillin-resistant *S. pseudintermedius* was isolated from the nares of veterinary workers and was identified as the cause of soft tissue infection in a human patient [21]. Lee et al. reported a case of NF in an immunocompromised human patient caused by *Streptococcus agalactiae*, *Arcanobacterium haemolyticum*, and *Finegoldia magna* after a minor bite from a dog [22]. The teeming numbers of microbes incriminated in NF cases necessitate microbial culture and sensitivity investigations in all suspected cases of NF to aid in targeted and effective antimicrobial therapies.

To the best of the authors' knowledge, this case is the first to report on NF in a dog with polymicrobial etiology due to *Macrococcus caseolyticus*, *Proteus mirabilis*, and *Escherichia coli*, without the involvement of the often-isolated methicillin-resistant *Staphylococcus pseudintermedius*. To what extent the roles of these three bacteria are in this case report merits further investigations.

## Figures and Tables

**Table 1:** Full blood count of the patient (dog).

Blood cell type	Result	Range	Unit
WBC	29.33	6.00–17.00	10^9^/L
Lymphocyte	2.15	0.83–4.69	10^9^/L
Monocyte	3.48	0.14–1.97	10^9^/L
Neutrophil	20.92	3.62–11.32	10^9^/L
Eosinophil	0.11	0.04–1.56	10^9^/L
Basophil	0.23	0.00–0.12	10^9^/L
PLT	290.00	117.00–460.00	10^9^/L
RBC	5.42	5.10–8.50	10^12^/L
HGB	13.90	11.00–19.00	g/dL
HCT	35.30	36.00–56.00	%
MCV	65.30	62.00–78.00	fL
MCH	25.60	21.00–28.00	Pg
MCHC	39.00	30.00–38.00	g/dL
RDW	8.00	14.50–19.20	%

**Table 2:** Serum biochemistry results.

Parameter	Result	Range	Unit
Potassium	4.28	3.50–5.20	mmol/L
Sodium	131.70	136.00–145.00	mmol/L
Chloride	92.00	96.00–108.00	mmol/L
Total protein	71.30	54.00–75.00	g/L
Albumin	25.40	23.00–31.00	g/L
Globulin	45.9	25.00–45.00	g/L
Urea	4.50	2.90–10.00	mmol/L
Creatinine	67.00	44.00–150.00	*μ*mol/L
Total bilirubin	9.40	2.00–5.13	*μ*mol/L
Direct bilirubin	3.59	1.03–2.05	*μ*mol/L
AST	180.04	9.00–48.50	U/L
ALP	398.80	1.00–114.00	U/L
ALT	68.68	10.00–109.00	U/L
GGT	15.00	3.00–19.00	U/L

**Table 3:** Bacterial culture and identification test results.

Isolates	Antimicrobial sensitivity test								Score value (Bruker MALDI Biotyper)
	Tet (10 *μ*g)	Cot (25 *μ*g)	Crx (30 *μ*g)	Gen (10 *μ*g)	Ery (15 *μ*g)	Flx (5 *μ*g)	Pen (15 *μ*g)	Amp (10 *μ*g)	
*Macrococcus caseolyticus*	++	++	+++	—	R	R	R	R	1.84
*Escherichia coli*	++	+	+++	+++	R	R	R	R	2.22
*Proteus mirabilis*	++	++	+++	—	R	R	R	R	2.14

## Data Availability

Underlying data can be obtained from the corresponding author (Dr. Dominic Osei).
